# Editorial: Improving Bayesian Reasoning: What Works and Why?

**DOI:** 10.3389/fpsyg.2015.01872

**Published:** 2015-12-02

**Authors:** David R. Mandel, Gorka Navarrete

**Affiliations:** ^1^Department of Psychology, York UniversityToronto, ON, Canada; ^2^Laboratory of Cognitive and Social Neuroscience, Psychology Department, UDP-INECO Foundation Core on Neuroscience, Universidad Diego PortalesSantiago, Chile

**Keywords:** Bayesian reasoning, belief revision, risk communication, subjective probability, human judgment, individual differences, probabilistic judgment

This edited collection was motivated by an interest in understanding how to improve Bayesian reasoning. In that sense, the book before you is pragmatically and prescriptively oriented. Several of the papers address that challenge and some pick up on the important question of why certain factors work as well as they do. However, *Improving Bayesian Reasoning: What Works and Why* offers more than its editors had bargained for or its title suggests. Many papers offer methodological and conceptual insights that should help readers understand the *psychology* of Bayesian reasoning as practiced in cognitive science.

The book is comprised of 23 papers by 48 authors. The contributions are ordered by type: 10 original research articles first, followed by three reviews and 10 shorter essays. Foregoing an attempt to summarize each contribution in sufficient detail, let us simply draw out some observations about the collection.

## Original research articles

This collection extends the base of original research on Bayesian reasoning in many important ways. Several papers offer further empirical evidence of the advantage of using visualized natural frequencies to communicate statistical information. Hoffrage et al. (2015b) show that the benefits of natural frequency representations in Bayesian tasks generalize from single- to multiple-cue cases and also to cases involving more than two hypotheses. Mandel (2015) shows that brief instruction in Bayesian reasoning using natural-frequency trees improves the coherence of intelligence analysts' posterior probability estimates. Binder et al. (2015) find that performance is improved when statistical information is communicated as natural frequencies instead of probabilities, and the natural-frequency format strengthens the facilitative effect of nested-set visualizations (i.e., tree diagrams and contingency tables) on Bayesian reasoning.

Other contributions identify where facilitative factors have their greatest impact. For instance, Hoffrage et al. (2015a) find that inexperienced business majors benefit more from natural-frequency formats than experienced business managers. Garcia-Retamero et al. (2015) address questions of *where* and *why* by showing that grid representations of natural frequencies facilitate Bayesian reasoning more strongly in medical patients with low numeracy, and that representational effects on reasoning are mediated by metacognitive judgment calibration. Hafenbrädl and Hoffrage (2015) go even further by parameterizing Bayesian skill using quantitative and qualitative factors that were free to vary across earlier studies. Finally, by triangulating choice and process data using an ecological sampling approach, Domurat et al. (2015) observe that many ostensibly Bayesian responses follow from use of an alternative statistical integration strategy.

The study of deduction had long been associated with reasoning from certain premises to certain conclusions. Yet Evans et al. (2015) and Cruz et al. (2015) venture into relatively new territory by examining the quality of reasoners' *uncertain* deductions using coherence-based Bayesian metrics such as probabilistic validity. These papers capture the fundamental insight that, even in deduction, most arguments consist of uncertain premises from which uncertain conclusions are drawn.

Finally, the contribution by Douven and Schupbach (2015) is pragmatic in two unique senses. First, it causes us to reconsider whether Bayesianism is the most appropriate normative framework in some contexts. Second, in the tradition of the great American pragmatist Charles Sanders Peirce, it situates abduction within the normative fold. The authors argue that explanationist alternatives to Bayesianism not only withstand normative critiques, they also fare better descriptively.

## Review articles and essays

The articles in this category draw out several dominant themes. First, the debate over natural frequencies vs. nested sets is passé. Although disagreement over the merits of the evolutionary account within which the original natural-frequency arguments were put forth linger, there is wide consensus that natural-frequency formats improve Bayesian performance by clarifying nested-set relations, which confers both representational and computational benefits (Brase and Hill, 2015).

Second, there has been a move away from the dual-systems account that emphasized System 1 sources of Bayesian error (Barbey and Sloman, 2007) toward a view that regards such errors as primarily due to representational and computational breakdowns in a problem-solving process, which occur even when explicit “System 2” processes are utilized (Johnson and Tubau, 2015; Sirota et al., 2015). For example, Juslin (2015) illustrates that Bayesian performance improves when computational requirements are shifted from multiplicative integration to additive integration. Likewise, Girotto and Pighin (2015) review studies showing that children and preliterate adults exhibit extensional reasoning that enables them to solve Bayesian problems provided they do not require explicit mathematical computation. The emerging view is further tempered by considerations of task characteristics, which are likely to alter the balance of implicit and explicit cognitive processes (Vallée-Tourangeau et al., 2015).

Whereas, most papers in this collection focus on Bayesian reasoners' performance, two refocus our attention on Bayesian communication by experts. Navarrete et al. (2014) make us consider how parents' decision-making about prenatal screening might be altered if they were given the positive predictive value (namely, the Bayesian value) of the initial screening test (which happens to be quite low) and also if parents received clear communications about the probabilistic risks of secondary invasive testing. Navarrete et al. (2015) generalize the argument, recommending that, where feasible, medical practitioners should give clients the relevant positive predictive values adjusted for their reference class. In short, clients should be relieved of computational burdens as far as possible so that they can focus on value-based decisions among available options.

Finally, several papers in this collection take the literature to task. Mandel (2014a) and McNair (2015) note that the definition of Bayesian reasoning in most psychological studies is mainly about information-integration performance. Few studies even require subjects to revise or update their beliefs! Others point to a lack of due attention to individual differences in reasoning and to the cognitive processes that lead to final estimates (Johnson and Tubau, 2015; McNair, 2015; Vallée-Tourangeau et al., 2015). Baratgin (2015) and Mandel (2014a) both take Bayesian researchers to task over their disregard of the subjectivist (and coherence-centered) foundations of Bayesianism.

However, attention to problems that have a temporal component is not lacking in this collection: Tubau et al. (2015) provide an insightful and comprehensive review of the Monty Hall Problem and Baratgin (2015) uses the two-player version of that problem to expose logical and terminological breakdowns in earlier theoretical analyses. Mandel (2014b) explores the perhaps even more complex Sleeping Beauty problem, which involves belief revision under conditions of asynchrony, to highlight how visual representations using quasi-logic trees can help clarify points of philosophical disagreement in the literature.

We hope readers will find this book informative, thought provoking, and of practical value.

## Author contribution

Both authors wrote the manuscript.

### Conflict of interest statement

The authors declare that the research was conducted in the absence of any commercial or financial relationships that could be construed as a potential conflict of interest.
